# Psyllium (*Plantago Ovata* Forsk) Husk Powder as a Natural Superdisintegrant for Orodispersible Formulations: A Study on Meloxicam Tablets

**DOI:** 10.3390/molecules24183255

**Published:** 2019-09-06

**Authors:** Gailute Draksiene, Dalia M. Kopustinskiene, Robertas Lazauskas, Jurga Bernatoniene

**Affiliations:** 1 Department of Drug Technology and Social Pharmacy, Faculty of Pharmacy, Medical Academy, Lithuanian University of Health Sciences, Kaunas 50161, Lithuania; 2 Institute of Pharmaceutical Technologies, Faculty of Pharmacy, Medical Academy, Lithuanian University of Health Sciences, Kaunas 50161, Lithuania; 3 Institute of Physiology and Pharmacology, Medical Academy, Lithuanian University of Health Sciences, Kaunas 50161, Lithuania

**Keywords:** orodispersible tablets, meloxicam, psyllium husk powder, disintegrant, dissolution rate, *in vitro* release

## Abstract

(1) Background: In this work, we investigated the application of a natural superdisintegrant, psyllium (*Plantago ovata* Forsk) husk powder, for the manufacture of orodispersible meloxicam tablets. Meloxicam was chosen as a model compound for the study. (2) Methods: The tablets were prepared using different concentrations of psyllium husk by direct compression. Bulk density, tapped density, hardness, friability, *in vitro* disintegration, and dissolution time tests were used to assess the quality of the formulations. (3) Results: Psyllium husk powder significantly increased the dissolution rate of meloxicam. The formulation containing 16 mg of psyllium husk powder showed the lowest wetting time, the highest water absorption ratio, and the lowest disintegration time compared to the control and to the other formulations. These effects may be attributed to the rapid uptake of water due to the vigorous swelling ability of psyllium husk powder. (4) Conclusions: The powder could be recommended as an effective natural superdisintegrant for orodispersible formulations.

## 1. Introduction

*Plantago ovata* belongs to the family of *Plantaginaceae* [1]. The seeds and psyllium husk of this plant are valuable sources of fibers and mucilage. Psyllium husk is used in the pharmaceutical industry as a laxative [1], to lower the glycemic index [2,3], and for the development of controlled-release formulations [4,5]. Due to quick water absorption, the weight of psyllium husk increases up to 10 times [6]. Hydrocolloids make up 10–30% of psyllium husk; these are water soluble polysaccharides that form mucilage layers when exposed to water. During hydrolysis, mucilage splits and polysaccharides, including xylose, arabinose, galacturonic acid, rhamnose, and galactose, are obtained [7,8,9]. These compounds are responsible for the disintegrative properties of psyllium husk and could be applied as natural disintegrants in drug manufacturing [1,6,10].

Tablet disintegration rate has a big impact on the characteristics of release of the active substance from the drug formulation. This rate depends on the properties of the disintegrants present in the tablet which are responsible for disintegration enhancement. Disintegrants are the substances or substance mixtures that facilitate decomposition and disintegration of a tablet or other drug formulations into smaller particles [11,12]. Thus, disintegrants are used to ensure that tablets split into smaller fragments during water contact, thereby achieving a faster disintegration rate [11,12]. Disintegrants improve the efficiency of solid drug formulations due to the decreased disintegration time that accordingly increases the dissolution rate and enhances the release of active substances [11,12,13,14]. Synthetic or semi-synthetic disintegrants are commonly used to manufacture tablets [10,15]. The main members of this group are croscarmellose sodium, sodium starch glycolate, and crospovidone, which is more compressible than other superdisintegrants, giving high breaking force and low friability. However, the disintegrants of natural origin have more advantages compared to synthetic substances [10,15], including that they are locally accessible, eco-friendly, bio-acceptable, renewable, and most cost-effective compared to synthetic products [16]. Furthermore, polysaccharide hydrocolloids, such as mucilage, gums, and glucans, are abundant in nature and, due to their high water-binding capacity, they could be used as swelling agents. There are only a few disadvantages of natural superdisintegrants, mainly that they are more hygroscopic, some of them are anionic, and they may cause *in vitro* binding [10,15].

Orodispersible tablets differ compared to standard tablets as they quickly disintegrate or disperse in the mouth before swallowing, causing a fast release of active compounds [11,14]. European pharmacopeia adopted the term orally disintegrating (orodispersible) tablet for a tablet that disperses or disintegrates in less than three minutes in the mouth before swallowing. Due to this short disintegration time, the bioavailability of active compounds from orodispersible tablets is much higher compared to other tablet types. Orodispersible tablets are a very convenient and widely used drug formulation for pediatric [17,18] and geriatric patients [17], pregnant women, patients with depression, or disabled patients with nausea, vomiting, or swallowing difficulties [14,19,20,21].

Meloxicam is a non-steroidal anti-inflammatory drug that has a highly variable bioavailability due to its poor aqueous solubility and dissolution [22]. The prolonged absorption with a *t*_max_ longer than 5 h indicates that the drug is slowly absorbed after oral administration [22,23]. To develop an oral formulation of meloxicam with a faster onset of action while maintaining the prolonged exposure, the increase in its aqueous solubility and enhancement of its dissolution are of great therapeutic importance [22,23]. Previous studies on orodispersible meloxicam tablets with microcrystalline cellulose and synthetic disintegrants, including croscarmellose sodium, sodium starch glycolate, and crospovidone, alone and in combination, showed a water absorption ratio of 71–78% and a drug release of ~70% after 60 min [24]. Since natural disintegrants are preferred due to their non-toxicity [16] and they generally reduce *in vitro* dissolution time and increase the water absorption ratio [25], in this study we investigated the application of the natural disintegrant psyllium (*Plantago ovata* Forsk) husk powder for the manufacture of orodispersible meloxicam tablets. Psyllium husk polysaccharide isolated by the solvent precipitation method was successfully used as superdisintegrant for valsartan orodispersible formulation [26], demonstrating an *in vitro* disintegration time of 20.34 s, a wetting time of 12.60 s, a water absorption ratio of 143.37, and a cumulative drug release of ~75% after 2 min. A cumulative drug release of nearly 100% after 8 min was reported for a 7.5% psyllium husk polysaccharide formulation [26]. Psyllium husk mucilage was used for prochlorperazine maleate fast disintegration tablets, demonstrating an *in vitro* dispersion time of 8 s, a wetting time of 11 s, and a water absorption ratio of 86%; a faster drug release (t_50%_ 3.3 min) was reported for the 8% psyllium husk mucilage formulation [6]. However, the use of psyllium husk itself without additional pre-processing could have more advantages in the reduction of tablet manufacturing time and costs.

In our study, the tablets were prepared using different concentrations of psyllium husk by direct compression. Bulk density, tapped density, hardness, friability, *in vitro* disintegration, and dissolution time tests were used to assess the quality of the formulations.

## 2. Results and Discussion

### 2.1. Composition of Orodispersible Meloxicam Tablets

**Figure 1:**
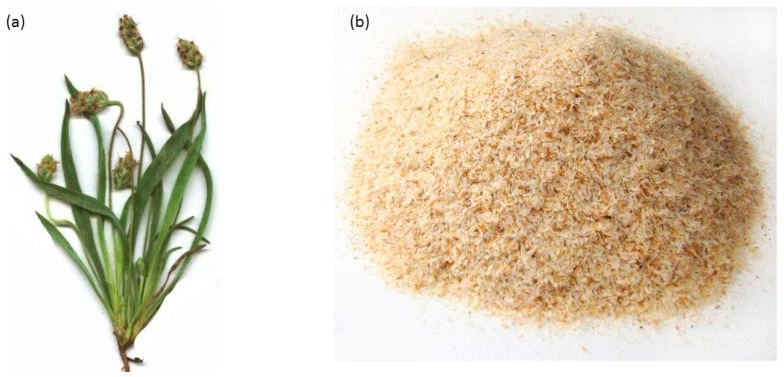
*Plantago ovata* (Forsk). (**a**) Plant; (**b**) psyllium husk powder.

Orodispersible meloxicam tablets were prepared by the direct compression method, which was the most simple and cost-effective technique [11,12]. Sorbitol, a highly compressible saccharide, was capable of ensuring excellent flowability and compressibility [27,28]; it also demonstrated a pleasant taste, therefore, it was chosen as a diluent along with mannitol. Mannitol has a negative heat of solution, which is responsible for a pleasant cooling sensation in the mouth. It also provides multidimensional benefits, such as good aqueous solubility and good wetting properties [29,30], that facilitate tablet breakdown. Psyllium (*Plantago ovata* Forsk) husk powder (Figure 1) was added as a natural superdisintegrant.

In our study, four formulation batches (M1–M4) with different psyllium husk powder amounts and one control batch (M5) without superdisintegrant addition were prepared (Table 1).

### 2.2. Pre-Compression Parameters of the Prepared Formulations

**Figure 2:**
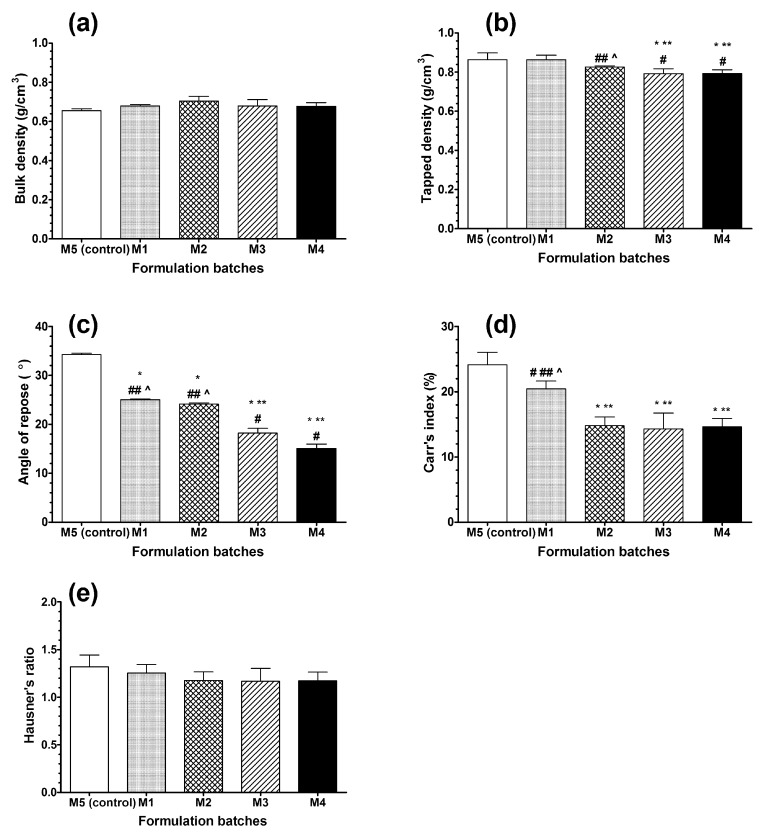
Pre-compression parameters: (**a**) Bulk density, (**b**) tapped density, (**c**) angle of repose, (**d**) Carr‘s index, and (**e**) Hausner‘s ratio of the prepared powder blends. Data are presented as the mean ± SD, n = 5. The results were analyzed with one-way analysis of variance (ANOVA) followed by Tukey’s multiple comparison test. The batches were compared to the control and to each other. *p* < 0.05 showed a statistically significant difference: * compared to control (M5), ** compared to M1, # compared to M2, ## compared to M3, ^ compared to M4.

Pre-compression parameters of all prepared formulation batches were evaluated in the next series of experiments (Figure 2a–e).

There was no significant difference in bulk density between the control and formulation batches (M1–M4) (Figure 2a), however, due to increasing particle size with higher amounts of psyllium husk in M3 and M4 batches, we noticed a slight, but statistically significant, decrease in tapped density (Figure 2b). It was known that angle of repose values <30° indicated “excellent” flow properties and values >56° indicated “very poor” flow properties. The intermediate values indicated “good” (31–35°) and “fair” (36–40°) flow properties [31]. The angle of repose of the formulation blend (M1, M2, M3, and M4) was in the range of 15.07–25.00° (Figure 2c), indicating excellent flow of the powder blend. The angle of repose of the control blend (M5) was 34.25°, indicating good flow. Lower Carr’s indexes indicated better powder flowability and a high Hausner ratio reflected a smaller capability to flow freely. The Carr’s indexes and Hausner’s ratios of the formulation blends (M1, M2, M3, and M4) were found to be in the range of 14.29–20.43 (Figure 2d) and 1.167–1.253 (Figure 2e), indicating good or medium flowability and good flowability, respectively, whereas the control blend (M5) showed acceptable flowability. While these techniques enabled a simple ranking of the flow behavior, the results could not be directly compared between techniques [32]. The values of the evaluated pre-compression parameters evalated were within the recommended limits of *European Pharmacopoeia* (Ph. Eur. 2.9.36.-2. Table of Scale flowability) and indicated excellent or good free flowing properties of the prepared powder blends (Figure 2a–e).

### 2.3. Post-Compression Parameters of the Prepared Formulations

**Figure 3:**
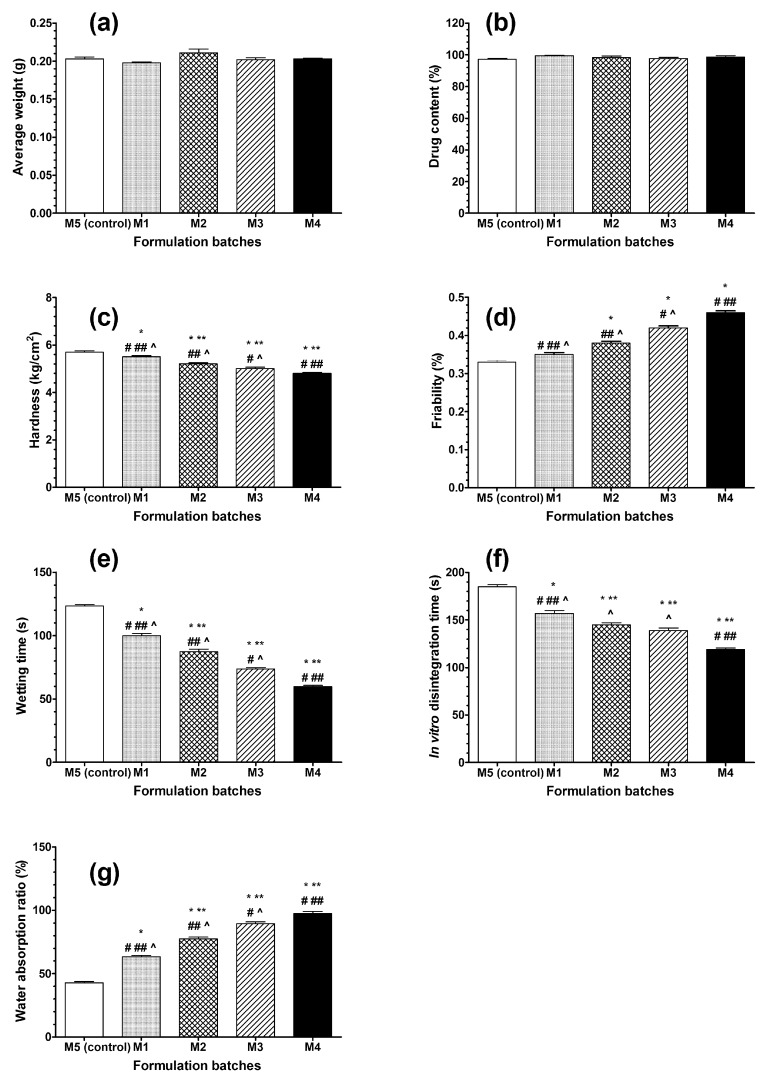
Post-compression parameters of orally disintegrating meloxicam tablets: (**a**) Average weight, (**b**) drug content, (**c**) hardness, (**d**) friability, (**e**) wetting time, (**f**) *in vitro* disintegration time, and (**g**) water absorption ratio. Data are presented as the mean ± SD, n = 5. The results were analyzed with one-way analysis of variance (ANOVA) followed by Tukey’s multiple comparison test. The batches were compared to the control and to each other. *p* < 0.05 showed a statistically significant difference: * compared to control (M5), ** compared to M1, # compared to M2, ## compared to M3, ^ compared to M4 batch.

After the powder blends were compressed into tablets, the post-compression parameters of all of the prepared formulations were evaluated (Figure 3a–g).

All tablets from each formulation passed a weight variation test (Figure 3a) as the percentage of weight variation was within the limits (± 7.5%) defined in the requirements of *European Pharmacopoeia* (Ph. Eur. 01/2016:20905). All tablets were of uniform appearance and the thickness varied from 3.8 to 3.84 mm. The drug content was found to be in the range of 97.7% to 99.5% (Figure 3b), which was within the acceptable limits (85–115%) described by *European Pharmacopoeia* (Ph. Eur. 01/2016:20906).

General problems encountered by rapidly disintegrating tablets are related to low physical resistance, low drug loading, and high friability [11,13,24]. A hardness test was used to determine the hardness of all tablet formulations. The hardness of the tablets was found to be in the range of 4.8–5.7 kg/cm2 (Figure 3c). A friability of less than 1% is required by *European Pharmacopoeia* (Ph. Eur. 01/2016:20907). In our study, the friability of all formulations was between 0.33–0.46% (Figure 3d), indicating that tablets had good mechanical resistance. Swamy et al. reported the hardness and friability of the optimized orodispersible meloxicam (7.5 mg) formulation with synthetic disintegrants (2% *w*/*w* sodium starch glycolate and 1.5% *w*/*w* croscarmellose sodium) to be 3.5 kg/cm^2^ and 0.69% [33]; thus, the psyllium husk was responsible for the slightly better physical resistance of the tablets compared to synthetic disintegrants.

The water absorption ratio and wetting time are important criteria in the understanding of the capacity of the disintegrants to swell in the presence of a small amount of water. The wetting time for all formulations was within the range of 59.67–123.33 s (Figure 3e). The formulation containing 16 mg of psyllium husk powder showed the lowest wetting time (59.67 s, Figure 3e) and the highest water absorption ratio (97.33%, Figure 3g) compared to other formulations containing psyllium husk powder. In a study conducted by Swamy et al., the wetting time of the optimized orodispersible meloxicam (7.5 mg) formulation with synthetic disintegrants (2% *w*/*w* sodium starch glycolate and 1.5% *w*/*w* croscarmellose sodium) were 5 s and 76.4%, respectively [33]. This wetting time, which was 10 times lower compared to our results, could be related to the different composition of the orodispersible tablets, i.e., microcrystalline cellulose in the study by Swamy et al. and mannitol in our study. However, the water absorption ratio was much higher using psyllium husk as a superdisintegrant compared to the synthetic compounds. A wetting time of 12–50 s was also reported in the investigation conducted by Pawar and Varkhade [26], but this cannot be directly compared to our work as another model compound, valsartan, with different solubility characteristics than meloxicam, was used in their study.

The *in vitro* disintegration time for all formulations varied from 119 s to 185 s (Figure 3f). The M4 formulation, which contained 16 mg of psyllium husk powder, showed the lowest disintegration time (119 s). The disintegration time of commercially available orodispersible meloxicam, Mobic^TM^ tablets (7.5 mg), which contain povidone as a disintegrant, was reported to be 210 ± 16.92 s [24]. Thus, psyllium husk could improve the disintegration time of orodispersible meloxicam formulations.

### 2.4. In Vitro Dissolution Studies

**Figure 4:**
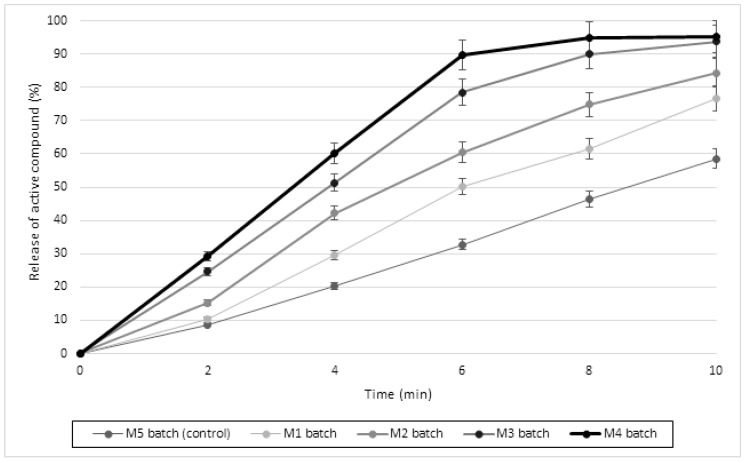
*In vitro* dissolution profile of orodispersible meloxicam tablets formulated with different amounts of psyllium (*Plantago ovata* Forsk) husk powder. Data are presented as the mean ± SD, n = 6. The results were analyzed with one-way analysis of variance (ANOVA) followed by Tukey’s multiple comparison test. M1–M4 batches significantly differed (*p* < 0.05) compared to the control (M5) and between themselves.

All formulations of the orodispersible meloxicam tablets were subjected to *in vitro* dissolution studies in phosphate buffer (pH 6.8) mimicking saliva pH to evaluate the superdisintegrating effect of the psyllium husk powder (Figure 4).

The release of meloxicam directly depended on the amount of psyllium husk powder in the prepared tablets (Figure 4). The highest meloxicam release (95.36% after 10 min) was obtained from the M4 batch tablets, which contained 16 mg of psyllium husk powder. Our results showed the lowest meloxicam release (76.68% after 10 min) from the M1 batch tablets, which contained 11.5 mg of psyllium husk powder. More than 75% of meloxicam was released after 10 min from all tablets containing psyllium husk powder (M1–M4), whereas only 58.5% of meloxicam was released from the control tablets (Figure 4). A faster dissolution rate in the presence of psyllium husk polysaccharide was also observed in the study of valsartan orodispersible formulations [26], and psyllium husk in the investigation of famotidine tablets [34]. However, the meloxicam release from Mobic^TM^ tablets (7.5 mg) with povidone as a disintegrant was reported to be 65% after 10 min, which showed a dissolution efficiency of 79.1 ± 3.24% [24]. The release of meloxicam from the optimized orodispersible meloxicam (7.5 mg) formulation with synthetic disintegrants (2% *w*/*w* sodium starch glycolate and 1.5% *w*/*w* croscarmellose sodium) in the study by Swamy et al. was 34.17% [33]. Thus, our results suggest that the demonstrated faster dissolution effect could be due to the disintegration properties of psyllium husk powder.

### 2.5. Stability Studies

The optimized formulations (M3 and M4) were subjected to stability studies according to the International Council for Harmonisation of Technical Requirements for Pharmaceuticals for Human Use (ICH) guidelines for six months. The tablets were stored as bulk material. Tablets were evaluated for physical appearance, friability, drug content (%), and disintegration time (Table 2). The tablets did not show any significant change during storage. Furthermore, *in vitro* dissolution tests were repeated after storage and no significant difference was obtained compared to the results before storage. Thus, it was concluded that the optimized tablets had good stability during their shelf life.

## 3. Conclusions

The dissolution rate of meloxicam was significantly enhanced in the orodispersible tablets prepared with psyllium (*Plantago ovata* Forsk) husk powder as a superdisintegrant. The formulation containing 16 mg of psyllium husk powder showed the lowest wetting time, the highest water absorption ratio, and the lowest disintegration time compared to control and to the other formulations containing psyllium husk powder. These effects may be attributed to the rapid uptake of water due to the vigorous swelling ability of psyllium husk powder. The gradual increase in friability by 0.1% in the tablets with the most psyllium husk powder was noted, however, this was much lower than the threshold (<1%) required by *European Pharmacopoeia* (Ph. Eur. 01/2016:20907). Therefore, psyllium husk powder could be recommended as an effective natural superdisintegrant for orodispersible formulations.

## 4. Materials and Methods

### 4.1. Materials

Health Plus 100% pure psyllium (*Plantago ovata* Forsk) husk was obtained from iHerb.com (Moreno Valey, CA, USA). Meloxicam was obtained from Iroko Pharms LLC, Philadelphia, PA, USA. Magensium stearate, manitol, and sorbitol were obtained from AppliChem GmbH, Darmstadt. Germany. Metanol was obtained from Bárta a Cihlář, Rožnov pod Radhoštěm, Czech Republic.

### 4.2. Preparation of Psyllium (Plantago Ovata Forsk) Husk Powder

The dried *Plantago ovata* seed husk was powdered by Retsch™ ZM 200 Model Ultra-Centrifugal Mill (Fisher Scientific UK Ltd., Loughborough, UK) with the Retsch FV 2703 rotor for 5 min. The 0.5 mm sieve set was used to separate the powder. The sieved powder was collected and checked for absence of foreign particles. This was preserved in an air-tight, dry container for further use.

### 4.3. Pre-Compression Parameters of Powder Blend

The flow properties of the powder are vital for the performance of the tablet. Hence, the flow properties of the powder were analyzed before compression into tablet form.

Bulk and tapped volumes (V_0_ and V_tapped_) were measured by the method from *European Pharmacopoeia* (Ph. Eur., USP) using a density tester (SVM 102, Erweka, Germany). The determined values were then used for the calculation of Hausner’s ratio and Carr’s index:

(1) $$\mathrm{Hausner}\;\mathrm{ratio}\;\left(\mathrm{HR}\right)=V_{0}/V_{\mathrm{tapped}},$$

(2) $$\mathrm{Carr}\;\mathrm{index}\;\left(\mathrm{CI}\right)=\frac{100\times \left(V_{0}-V_{\mathrm{tapped}}\right)}{V_{0}}.$$

Flowability was evaluated according to Carr’s index and Hausner’s ratio (Ph. Eur. 2.9.36.-2. Table of Scale flowability).

The angle of repose was determined using the funnel method. The blend was poured through a funnel (105 mm in diameter, 190 mm high, with a stem 105 mm long, and an internal diameter of 5 mm) that could be raised vertically until a maximum cone height (h) was obtained. The radius of the heap (r) was measured and the angle of repose (θ) was calculated:

(3) $$\mathrm{Tan}\theta =\frac{h}{r}$$

### 4.4. Formulation of Orodispersible Tablets by Direct Compression Method

Orally disintegrating tablets were prepared using the direct compression method. All ingredients were passed through mesh No. 60 separately, then, the ingredients were weighed and mixed in geometrical order and compressed into tablets of 200 mg using 9 mm round flat punches on a manual hydraulic press (Specac Limited, Kent, UK). The mixing time was 10 min and the amplitude was 40 mm. A batch of 100 tablets was prepared for each of the designed formulations.

### 4.5. Post-Compression Parameters of Orally Disintegrating Tablets

All tablets were evaluated for different parameters, including appearance, weight variation, hardness, thickness, friability, wetting time, water absorption ratio, and disintegration time.

Weight variation tests were performed by weighing 20 tablets individually, calculating the average weight, and comparing the individual tablet weight to the average weight.

For drug content assay, 10 tablets were weighed and carefully grinded to receive a homogenous powder. The powder was dispersed in 10 mL of methanol solution in a volumetric flask and kept for 15 min in an ultrasound bath (Memmert WNB7 waterbath, Memmert GmbH & Co. KG, Schwabach, Germany). The solution was passed through a 0.22 µm membrane filter for UPLC analysis. Determination of the meloxicam content was carried out using the Waters Acquity UPLC chromatography system (Waters, Milford, MA, USA), which was equipped with a photodiode array detector (Waters 996 UPLC) at a wavelength of 350 nm.

The tablet friability test was performed using the SOTAX FT2 tablet friability tester (Sotax GmbH, Lörrach, Germany). A total of 20 tablets were weighed with an accuracy of 0.001 g and transferred to the tester. The internal diameter of the drum was 200 mm, the rotation speed was 25 ± 1 rpm, and the testing time was 5 min. At each turn of the drum, the tablets rolled or slid and fell onto the drum wall or onto each other. After the test, the tablets were removed and accurately weighed. Tablet friability (D) was calculated according to the formula:

(4) $$D=\frac{m_{1}-m_{2}}{m_{1}}\;\times 100\text{\%},$$

where D is friability (%), m_1_ is the initial tablet weight (g), and m_2_ is the tablet weight after the friability test (g).

Tablet hardness was determined using the manual tablet hardness tester SOTAX HT1 (Sotax GmbH, Lörrach, Germany).

For a wetting time assay, a tablet was transferred onto a piece of tissue paper of 10 cm diameter, which was placed in a Petri dish with a 10 cm diameter containing 10 mL of water. The time required for water to reach the upper surface of the tablet was been noted as the wetting time.

The water absorption test was performed using the same procedure as that of the wetting time. A tablet was weighed and then transferred onto a piece of tissue paper of 10 cm diameter, which was placed in a Petri dish with a 10 cm diameter containing 10 mL of water. The tablet was weighed again after the water reached the upper surface of the tablet. The water absorption ratio (R) was calculated according to the formula:

(5) $$R=\frac{V_{b}-V_{a}}{V_{a}}\times 100\%,$$

where V_a_ is the tablet weight before water absorption and V_b_ is the tablet weight after water absorption.

To assess the tablet disintegration time, 6 tablets were placed in 900 mL of distilled water in the basket rack of the SOTAX DT-2 disintegration tester (Sotax GmbH, Lörrach, Germany). One tablet was placed in each of the six tubes of the basket and the assembly was raised and lowered at a rate of 30 cycles per minute in the water at 37 ± 0.5 °C.

### 4.6. In Vitro Release Studies

Dissolution profiles of meloxicam in designed formulations of orodispersible tablets were determined using the SOTAX AT7 smart model semi-automated paddle type dissolution tester (Sotax GmbH, Lörrach, Germany). The basket method was applied using phosphate buffer (50 rpm, 700 mL). The pH value was maintained at 6.8 (37 ± 0.5 °C). Aliquots (5 mL) were manually extracted from parallel dissolution vessels at 2, 4, 6, 8, and 10 min time points, filtered through a membrane filter (0.22 µm), and quantified via UPLC with a photodiode array detector (Waters 996 UPLC) at a wavelength of 350 nm. The dissolution media in each vessel was topped off with fresh phosphate buffer (5 mL) to restore the original volume. The mean value of six trial runs and the standard deviation were calculated.

### 4.7. Stability Studies

Accelerated stability testing studies were performed for 6 months according to ICH guidelines at a temperature of 40 ± 2 °C and a relative humidity of 75 ± 5% in a CLIMACELL stability chamber (MMM Medcenter Einrichtungen GmbH, Planegg/München, Germany). The tablets were stored as bulk material. The samples were withdrawn after 0, 3, and 6 months of storage. Physical appearance assessment, tablet friability, drug content (%), and disintegration time tests were performed to evaluate the influence of storage on the stability of the tablets.

### 4.8. Statistical Analysis

Data are presented as the mean ± SD. Statistical analysis was performed by one-way analysis of variance (ANOVA) followed by Tukey’s multiple comparison test with the software package Prism v. 5.04 (GraphPad Software Inc., La Jolla, CA, USA). A value of *p* < 0.05 was used as the level of statistical significance.

## Figures and Tables

**Table 1:** The composition of orally disintegrating meloxicam tablets.

Ingredient (mg)	Formulation Code				
	M1	M2	M3	M4	M5
Meloxicam	7.5	7.5	7.5	7.5	7.5
Psyllium (*Plantago ovata* Forsk) husk powder	11.5	13.0	14.5	16.0	–
Sorbitol	169.0	167.5	166.0	164.5	180.5
Mannitol	10	10	10	10	10
Mg stearate	2	2	2	2	2
Total weight	200	200	200	200	200

**Table 2:** Stability data of optimized formulations at 40 °C and a relative humidity of 75%.

Formulation Code	Evaluation Parameters	Duration (months)		
		0	3	6
M3	Physical changes	No changes	No changes	No changes
	Friability (%)	0.42 ± 0.01	0.43 ± 0.01	0.45 ± 0.01
	Drug content (%)	97.7 ± 0.69	97.4 ± 0.70	97.0 ± 0.71
	Disintegration time (s)	139	138	138
M4	Physical changes	No changes	No changes	No changes
	Friability (%)	0.46 ± 0.01	0.49 ± 0.01	0.5 ± 0.01
	Drug content (%)	98.7 ± 0.82	98.7 ± 0.82	98.2 ± 0.80
	Disintegration time (s)	124	123.5	125
